# Champaign County Medical Society

**Published:** 1868-05

**Authors:** Sam’l H. Birney

**Affiliations:** Secretary


### CHAMPAIGN COUNTY MEDICAL SOCIETY.

---

The Fourth Regular Session of the Association was held at the office of Dr. C. H. Mills, in Champaign City, on Wednesday, April 1st, 1868, and was called to order by the President.

The minutes of the last meeting were read and approved.

A very interesting paper was read, on the use of the speculum in the treatment of uterine diseases, which was discussed with spirit and profit.

The following officers have been elected for the ensuing year:

*President*—Dr. C. H. MILLS.
*Senior Vice-President*—H. SOMERS.
*Junior* " " W. R. EARHART.
*Treasurer*—I. T. PEARMAN.
*Secretary*—S. H. BIRNEY.
*Censors*—H. C. HOWARD, I. MCHUGH, M. S. BROWN.
*Delegates to Illinois State Medical Association*—Drs. W. EARHART and S. H. BIRNEY.

On motion, the Secretary was directed to prepare an account of the proceedings of the Association for publication, and send copies to the Chicago *Medical Journal* and EXAMINER.

On motion, the Society adjourned, to meet in Urbana the first Wednesday in June.

I am happy to state that the Society is in a very flourishing condition.

SAM'L H. BIRNEY, *Secretary.*

